# Gaps in beliefs and practice in dyslipidaemia management in Japan, Germany, Colombia and the Philippines: insights from a web-based physician survey

**DOI:** 10.1186/s12944-020-01265-z

**Published:** 2020-06-10

**Authors:** Philip J. Barter, Shizuya Yamashita, Ulrich Laufs, Alvaro J. Ruiz, Rody Sy, Mark David G. Fang, Emanuela Folco, Peter Libby, Yuji Matsuzawa, Raul D. Santos

**Affiliations:** 1 School of Medical Sciences, University of New South Wales, Sydney, Australia; 2 Rinku General Medical Center and Osaka University Graduate School of Medicine, Osaka, Japan; 3 Universitatsklinikum Leipzig, Leipzig, Germany; 4 Pontificia Universidad Javeriana, Bogota, Colombia; 5 College of Medicine, University of the Philippines-Manila, Manila, the Philippines; 6 Cardinal Santos Medical Center, San Juan City, MetroManila, the Philippines; 7 International Atherosclerosis Society, Viale Piave 35, Milan, Italy; 8 Brigham and Women’s Hospital, Harvard Medical School, Boston, USA; 9 Sumitomo Hospital, Osaka, Japan; 10 Hospital Israelita Albert Einstein, São Paulo, Brazil; 11 Heart Institute -InCor, University of São Paulo Medical School Hospital, Av Dr Enéas C. Aguiar 44, Sao Paulo, SP CEP-05403-900 Brazil

**Keywords:** Low-density lipoprotein cholesterol, Atherosclerotic cardiovascular disease, Statins, Safety, Haemorrhagic stroke, Chronic kidney disease

## Abstract

**Background:**

Implementing evidence-based management of dyslipidaemia is a challenge worldwide.

**Objectives:**

To understand physician beliefs and behaviour and identify uncertainties in dyslipidaemia management across four world regions.

**Methods:**

Web-based survey of 1758 physicians in Japan, Germany, Colombia and the Philippines who were selected randomly from existing databases. Key inclusion criteria were 1) for cardiologists and diabetes/endocrinology specialists: ≥50 dyslipidaemia patients examined in the last month; 2) for specialists in neurology/neurosurgery/stroke medicine: ≥50 dyslipidaemia patients and ≥ 20 patients with a history of ischaemic stroke examined in the last month; and 3) for specialists in nephrology and general medicine: based at centres with ≥20 beds and ≥ 50 dyslipidaemia patients examined in the last month. The self-report survey covered dyslipidaemia management, target low-density lipoprotein cholesterol (LDL-C) levels in different patient groups, and statin safety. All physicians gave voluntary consent and all data were anonymised. Analysis was solely descriptive.

**Results:**

The survey highlighted key areas of uncertainty in dyslipidaemia management in the four countries. These related to LDL-C targets in different patient groups, the safety of low LDL-C levels, the safety of statins, especially for effects on cognitive, renal and hepatic function and for haemorrhagic stroke risk, and lipid management strategies in patients with chronic kidney disease, including those with concomitant hypertriglyceridaemia.

**Conclusions:**

This survey of physicians in Japan, Germany, Colombia and the Philippines has identified key gaps in knowledge about dyslipidaemia management. These relate to the safety of low LDL-C levels, the safety of statins, and lipid management of chronic kidney disease. The findings from this survey highlight the need for further education to improve the implementation of guideline recommendations for dyslipidaemia management.

## Introduction

Extensive and robust evidence has established low-density lipoprotein cholesterol (LDL-C) as causal for atherosclerotic cardiovascular disease (ASCVD) [1]. Irrespective of therapeutic strategy, lowering LDL-C levels reduces the risk of ASCVD events, as demonstrated in major cardiovascular outcomes studies in very high-risk patients treated with a statin [2] or non-statin therapy (i.e. ezetimibe or proprotein convertase subtilisin/kexin type 9 [PCSK9] inhibitors) [3–5]. The safety of these LDL-lowering therapies has also been demonstrated [2–5]. Despite this overwhelming body of evidence, controversy persists regarding the role of LDL-C as a cause of ASCVD. Frequent, non-evidence-based assertions published in the media suggest that statins are unsafe and that lowering LDL-C to very low levels is dangerous [6]. There is also uncertainty regarding the veracity of adverse effects of statins, including statin-associated muscle symptoms [7, 8]. Similarly, safety concerns have been raised regarding novel therapies, including the PCSK9 inhibitors [9].

The World Heart Federation has developed a series of ‘roadmaps’ which aim to reduce cardiovascular disease in developing world regions. One of these roadmaps has focused on identifying barriers to effective cholesterol management gaps in knowledge and practice [10]. There is little information, however, regarding the beliefs and behaviour of physicians responsible for managing patients with dyslipidaemia in their routine practice. To address this issue, an online survey was conducted in Japan in 2017 by the Japan Atherosclerosis Society in collaboration with the International Atherosclerosis Society (IAS) to determine the attitudes and practice of physicians responsible for lipid management [11]. Subsequent to this, a second survey in Japan and surveys in Colombia, Germany and the Philippines were conducted by the IAS. These aimed to evaluate cultural differences among physicians in their beliefs and routine practice of managing dyslipidaemia.

## Methods

This study was designed as a web-based survey, using an online questionnaire. The project was coordinated by the IAS. The IAS convened a committee, chaired by PB, RS, PL, SY, RDS, AR and UL, which was responsible for developing and implementing the survey in each country.

Physicians were selected randomly from existing databases in each country. In Japan, physician recruitment was conducted by CareNet, Inc., an online Japanese-language medical information service for physicians. All prospective participants were registered members of CareNet, Inc., and received an email introducing the study and inviting them to participate. In Germany, physicians were selected from a market research panel of approximately 17,000 doctors. In Colombia, a local healthcare fieldwork partner recruited physicians via e-mail and telephone. Finally, in the Philippines, physicians were recruited from a database created from an online ‘e-survey’ of internists, cardiologists and vascular specialists attending annual clinical conventions, or by questionnaires distributed at a workshop for cardiologists, and a local chapter convention for internists. In all countries, physicians who met the following criteria were accepted for inclusion in the survey: (i) expertise in cardiology, diabetes or endocrinology and treating ≥50 patients with dyslipidaemia in the previous month; (ii) expertise in neurology, neurosurgery or stroke medicine and treating ≥50 patients with dyslipidaemia and ≥ 20 patients with a history of ischaemic stroke in the previous month; and (iii) expertise in nephrology and general internists based at hospitals with ≥20 beds and treating ≥50 patients with dyslipidaemia in the previous month. All physicians gave voluntary consent before participation.

The study used a self-report web-based survey, which required 15–30 min for completion (Table 1). Briefly, the survey comprised 23 multiple choice questions that aimed to investigate beliefs and behaviour in the management of dyslipidaemia. These included questions relating to the role of LDL-C in ASCVD, target LDL-C levels in different patient groups, safety issues relating to low LDL-C levels and statin use, awareness and management of familial hypercholesterolaemia (FH), and current practice for the management of hypertriglyceridaemia in patients with chronic kidney disease (CKD). Data were anonymised and analysed descriptively by the authors. Categorical data were described as absolute numbers and percentages. No formal statistical analyses comparing different countries or different medical specialties were performed.

**Table 1:** Web-based survey used to investigate beliefs and behaviour in dyslipidaemia management in the four countries*

Question^a^	Responses
2. Do you believe that elevated LDL cholesterol is an important cause of coronary disease and ischaemic stroke?	Yes/No/Uncertain
3. Concerning use of statin, do you have concerns related to any of the following. (More than one item can be selected)?	· Increase of the risk of haemorrhagic stroke · Increase of the risk of cognitive impairment · Increase of the risk of new onset diabetes · Development of muscle disorder · Increased risk of hepatic disease · Others (Please specify) · Do not have any concern
4. Do you have concerns about lowering LDL cholesterol levels in patients with	· Haemorrhagic stroke: Yes/No/Uncertain · Ischaemic stroke: Yes/No/Uncertain · Transient ischaemic attack (TIA): Yes/No/Uncertain · Subarachnoid haemorrhage: Yes/No/Uncertain
5. Do you think statins have any effect on cognitive function?	Yes/No/Uncertain
6. Please indicate the percentage of patients who cannot use statins continuously due to adverse effects (such as muscle symptoms, etc.).	0% (I have no statin-intolerant patients) ≥ 0.1 to < 5% ≥ 5 to < 10% ≥ 10 to < 15% ≥ 15 to < 20% ≥ 20%
7. Please indicate your target level of LDL cholesterol after initiation of drug therapy in the following patient groups	· A history of any coronary artery disease: The target level of LDL cholesterol should be < mg/dl (please specify) · Without a history of coronary artery disease but with a history of diabetes mellitus/chronic kidney disease/ischaemic stroke/peripheral artery disease: The target level of LDL cholesterol should be < mg/dl (please specify) · Without a history of the conditions listed above: The target level of LDL cholesterol should be < mg/dl (please specify)
8. Do you have concerns about safety if the LDL cholesterol is below the following levels?	· 20 mg/dL (0.52 mmol/L) · 30 mg/dL (0.78 mmol/L) · 40 mg/dL (1.03 mmol/L) · 50 mg/dL (1.29 mmol/L) · 60 mg/dL (1.55 mmol/L) · 70 mg/dL (1.80 mmol/L) · 0ther value [mg/dL or mmol/L] · Does not have any opinion
9. Do you think markedly low LDL cholesterol levels affect the incidence of haemorrhagic stroke?	Yes/No/Uncertain
10. How much does the LDL cholesterol level affect the risk of inducing atherosclerotic cardiovascular diseases?	· Affects the risk significantly · Affects the risk moderately · Uncertain · Affects the risk to a small extent · Does not affect the risk
11 Do you sometimes use “non-HDL cholesterol level” as a risk index of atherosclerotic cardiovascular diseases (ASCVD, coronary artery diseases, non-cardiogenic cerebral infarction) or a therapeutic efficacy index during your medical practice?	· non-HDL cholesterol level is not used · non-HDL cholesterol level is sometimes used as “a risk index of ASCVD” · non-HDL cholesterol level is sometimes used as “a therapeutic efficacy index.” · non-HDL cholesterol level is sometimes used as both “a risk index of ASCVD” and “a therapeutic efficacy index.”
12. For Japan: Concerning “Comprehensive risk management chart for the prevention of cerebro- and cardiovascular diseases” developed in 2015 mainly by The Japanese Society of Internal Medicine, please inform us about the status of your recognition/use of the chart.	· I know about this chart and am actually using it · I know about this chart, but have never used it · I do not know about this chart.
For Germany: Concerning the European Guidelines (ESC/EAS) for lipid management.	· I know about the guidelines and I am actually using them · I know about the guidelines, but I have never used them. · I do not know about the guidelines
For Colombia: Concerning the AHA/ACC Guidelines for lipid management	· I know about the guidelines and I am actually using them · I know about the guidelines, but I have never used them. · I do not know about the guidelines
13. Concerning Familial Hypercholesterolaemia (FH, one type of primary hyperlipidaemia), which best reflects your practice?	· I know about FH and have patients with FH (which was found by my diagnosis) and am engaged in their treatment. · I know about FH and have referred patients with suspected FH to some other medical institution/physician. · I know about FH but have never seen a patient with suspected FH. · I do not know about FH.
14. When you make a diagnosis of FH in an adult patient (15-year-old or older), do you perform the followings? *(More than one item can be selected)*	· Palpation of Achilles tendon · X-ray photography of Achilles tendon · Take a family history of hyper-LDL-cholesterolaemia · Take a family history of FH · Take a family history of premature coronary artery diseases · None of the above
15. Do you think patients with FH have an increased incidence of ischaemic stroke?	Yes/No/Uncertain
16. Do you think statins have any adverse effects on renal function?	Yes/No/Uncertain
17. Do you think the lowering of LDL cholesterol reduces ASCVD events in patients with CKD?	Yes/No/Uncertain
18. If yes, is LDL cholesterol lowering therapy effective for patients with any stage of CKD?	Yes/No/Uncertain
19. What do you think is the target LDL cholesterol level for primary prevention of the patients with CKD?	< 140 mg/dL (< 3.62 mmol/L) < 120 mg/dL (< 3.10 mmol/L) < 100 mg/dL (< 2.6 mmol/L) < 70 mg/dL (< 1.8 mmol/L) Medicate without setting the target LDL cholesterol level
20. Do you think the target LDL cholesterol level is different between patients with different CKD stage?	Yes/No/Uncertain
21. Do you think there is a clinical benefit to treat CKD patients with hypertriglyceridaemia?	Yes/No/Uncertain
22. How do you treat CKD patients with hypertriglyceridaemia?	· Use statins · Use fibrates · Use nicotinic acid derivatives · Use n-3 polyunsaturated fatty acid · Manage through lifestyle modification without medications
23. Do you reduce the dose of statins in patients with CKD?	Yes/No/Uncertain

^a^Question 1 confirmed eligibility to participate in the survey: i.e. Concerning the patients you examined for the latest one month, please inform us the numbers of the followings

Number of patients with dyslipidaemia

Number of patients with a history of ischaemic stroke

Number of patients with (or with a history of) coronary heart disease

The number of patients you examined

The number of patients receiving drug treatment for dyslipidaemia

## Results

A total of 1758 physicians, 508 in Japan, 500 in Germany, 345 in Colombia, and 405 in the Philippines, took part in the survey. Across all four countries, most respondents were either general physicians (33%) or cardiologists (22%) (Table 2). Results are summarised below for findings that show either agreement or uncertainty between countries.

**Table 2:** Number of eligible physicians participating in the survey, summarised by country and specialty

	Japan	Germany	Colombia	Philippines	Total (%)
Specialty					
Cardiologists	103	100	55	122	380 (22%)
Endocrinologists	103	100	40	42	285 (16%)
Neurologists	102	100	70	7	279 (16%)
Nephrologists	100	100	20	12	232 (13%)
General physicians	100	100	160	222	582 (33%)
Total	508	500	345	405	1758

### Agreement between countries

**Fig. 1:**
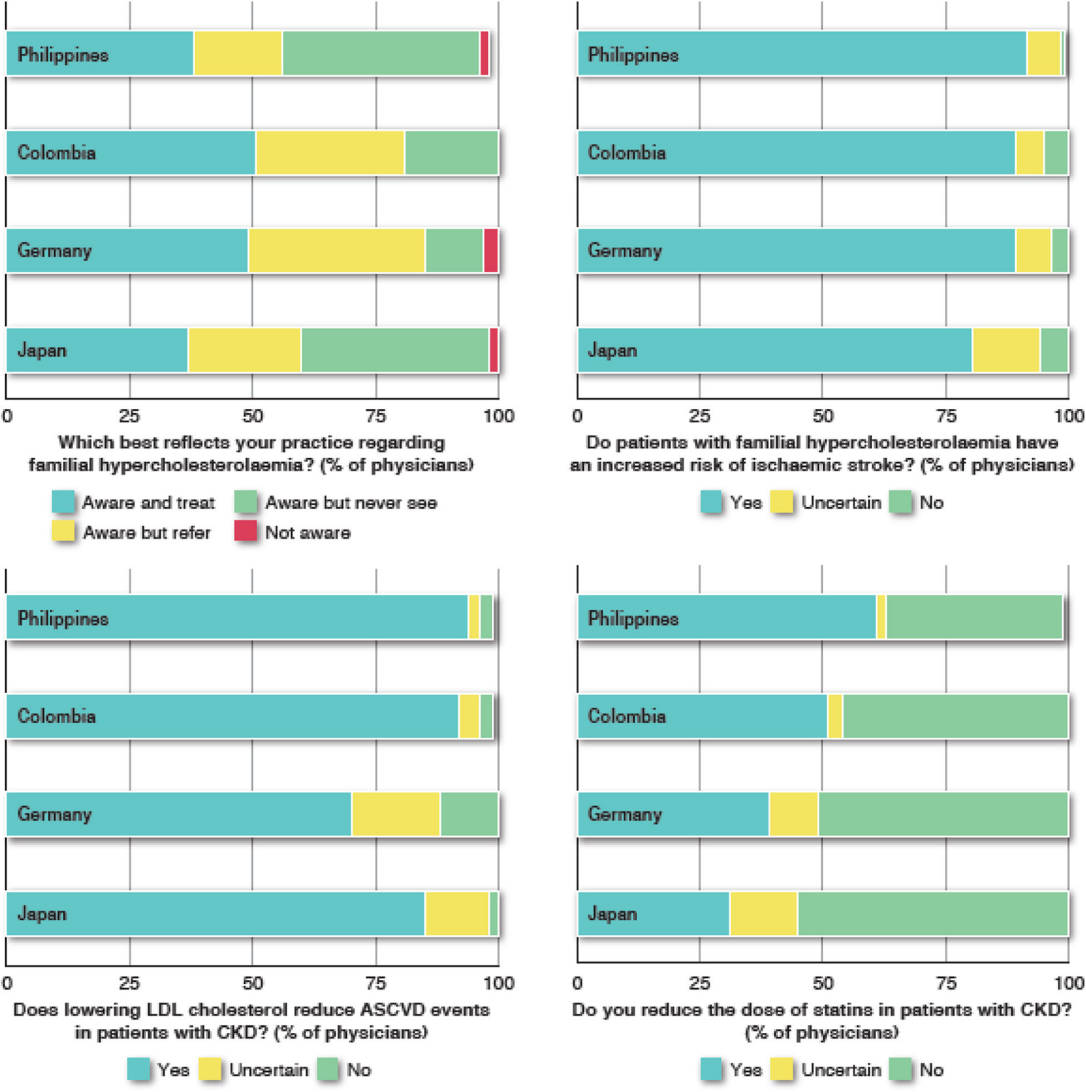
Agreement between physicians in Japan, Germany, Colombia and the Philippines for survey questions relating to familial hypercholesterolaemia and the management of LDL cholesterol in chronic kidney disease (CKD). Abbreviations: ASCVD atherosclerotic cardiovascular disease; LDL low-density lipoprotein

There was universal agreement in all four countries (95–99% of respondents) that an elevated LDL-C level is an important cause of coronary disease and ischaemic stroke. Recent efforts aimed at educating clinicians about FH have improved awareness, as > 95% of respondents in all four countries were aware about FH, and between 56% (in the Philippines) and 85% (in Germany) of physicians either treated FH patients or referred them to a specialist centre. Most physicians (81–92%) believed that FH patients were also at increased risk of ischaemic stroke (Fig. 1).

There was also agreement between all four countries that lowering LDL-C reduces the risk of ASCVD events in CKD patients (ranging from 70% of respondents in Germany to 94% in the Philippines) (Fig. 1). Most respondents believed that there was a benefit from using statins at any stage of CKD. Across the countries, between 31% (in Japan) and 61% (in the Philippines) of respondents believed that the statin dose should be reduced in patients with CKD (Fig. 1).

### Uncertainty between countries

The survey identified several areas of uncertainty in dyslipidaemia management, both within and across the four countries.

#### Target LDL-C levels

In people with established coronary heart disease, the target LDL-C level proposed by physicians varied, from 2.48 mmol/L (96 mg/dL) in Japan, 2.22 mmol/L (86 mg/dL) in Germany, 2.10 mmol/L (85 mg/dL) in the Philippines, to 2.07 mmol/L (80 mg/dL) in Colombia. Respondents in three countries believed that the target LDL-C level should be higher in people with diabetes, CKD or peripheral artery disease but without coronary heart disease (2.84 mmol/L [110 mg/dL] in Japan, 2.43 mmol/L [94 mg/dL] in Germany, and 2.25 mmol/L [87 mg/dL] in Colombia). In primary prevention patients without these conditions, the proposed target LDL-C levels were higher, ranging from 3.39 mmol/L (131 mg/dL) in Japan, 3.34 mmol/L (129 mg/dL) in Germany, 2.97 mmol/L (115 mg/dL) in Colombia and 2.72 mmol/L (105 mg/dL) in the Philippines. In Japan, Germany and the Philippines less than half of the respondents, versus 75% in Colombia, used non-high-density lipoprotein cholesterol either to determine global risk or as a therapeutic target.

#### Management of CKD

Beliefs regarding the target LDL-C level in CKD patients varied between the four countries. In Japan, 67% of respondents believed that the target LDL-C should be < 3.1 mmol/L (120 mg/dL). In the other three countries, between 40% (in the Philippines) and 57% (in Colombia) believed that the target LDL-C should be < 2.6 mmol/L (100 mg/dL), and between 21% (in Germany) and 27% (in Colombia and the Philippines) believed that the target LDL-C should be < 1.8 mmol/L (70 mg/dL). The perceived clinical benefit of treating CKD patients with hypertriglyceridaemia also varied between countries, from 91% in Colombia to 42% in Germany. While the pharmacotherapeutic options for hypertriglyceridaemia in CKD were similar in the four countries (statins, fibrates and omega-3 fatty acids), 30% of physicians in Germany believed that lifestyle modification alone was the most appropriate treatment for CKD.

#### Safety of low LDL-C levels

About half of the respondents in the four countries had concerns about the safety of low LDL-C levels (≤1.29 mmol/L or ≤ 50 mg/dL) (Table 3). The risk for haemorrhagic stroke with low LDL-C levels was a key concern, although there was also uncertainty (40% of clinicians in Japan and Germany, 38% in Colombia and 44% in the Philippines were uncertain about this issue).

**Table 3:** Percentage of physicians with concerns about safety below the following LDL-C levels, summarised by country

	Japan	Germany	Colombia	Philippines
LDL-C level mmol/L (mg/dL)		(% of physicians)		
< 0.52 (20)	2	13	10	13
< 0.78 (30)	8	10	8	10
< 1.03 (40)	16	11	14	8
< 1.29 (50)	23	14	14	13
< 1.55 (60)	17	5	22	7
< 1.80 (70)	7	7	12	9
Other	1	3	2	0
No opinion	26	38	26	34

#### Statin safety

**Fig. 2:**
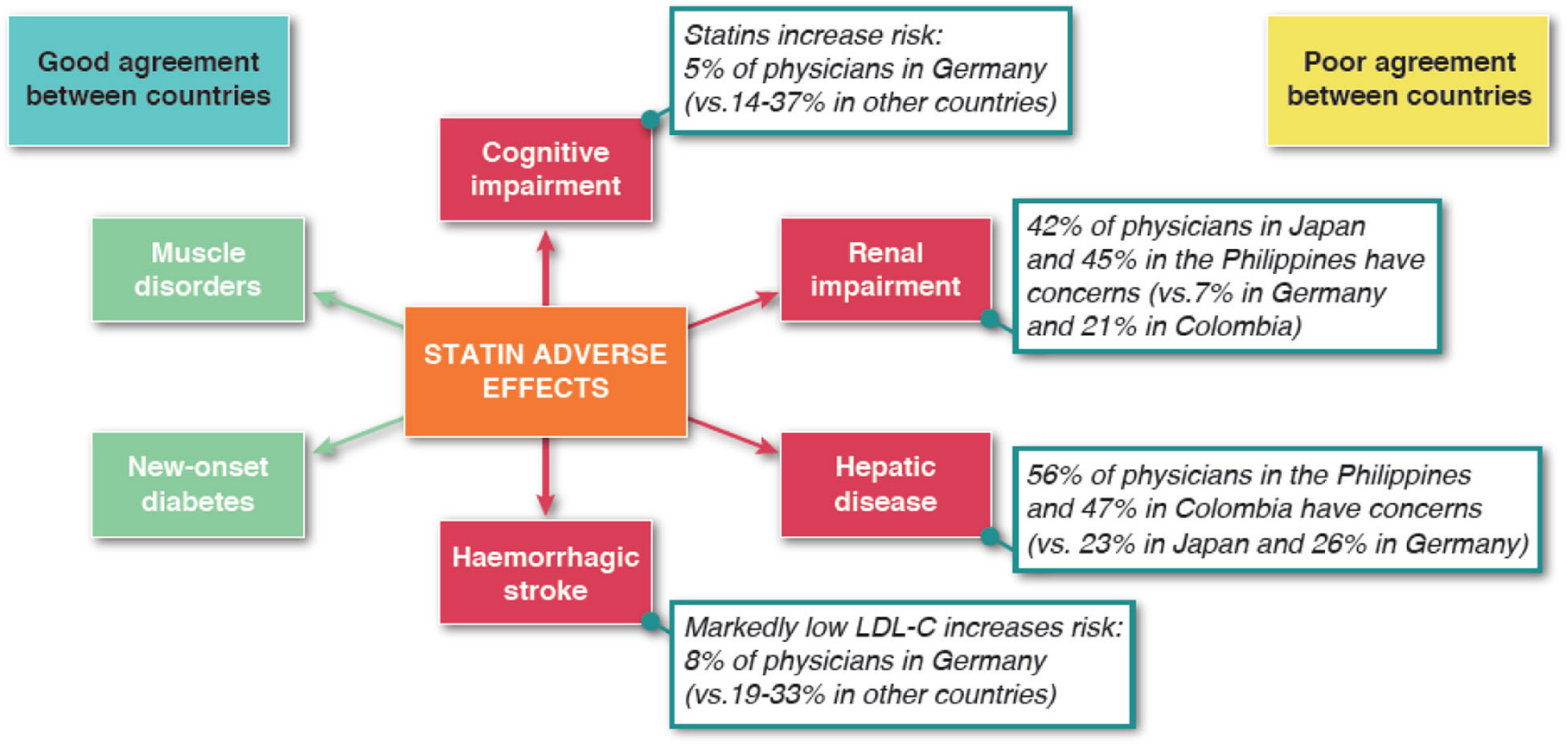
Agreement and disagreement between physicians in Japan, Germany, Colombia and the Philippines relating to the adverse effects of statin therapy. Abbreviations: LDL-C low-density lipoprotein cholesterol

Physicians in all four countries recognised that a small proportion of patients are unable to use statins continuously due to adverse effects. Estimates of the percentage of affected patients varied from < 5% reported by most respondents in Japan, Colombia and the Philippines, to 5–15% reported by half of respondents in Germany. The survey highlighted uncertainty in all four countries regarding the effects of statins on cognitive, renal, and hepatic function (Fig. 2). A substantial proportion of respondents in each of the four countries was uncertain whether statins adversely affect cognitive function (24% in Germany, 26% in Colombia, 36% in the Philippines and 42% in Japan), or renal function (16% in Germany, 8% in Colombia, 13% in the Philippines and 36% in Japan).

## Discussion

**Fig. 3:**
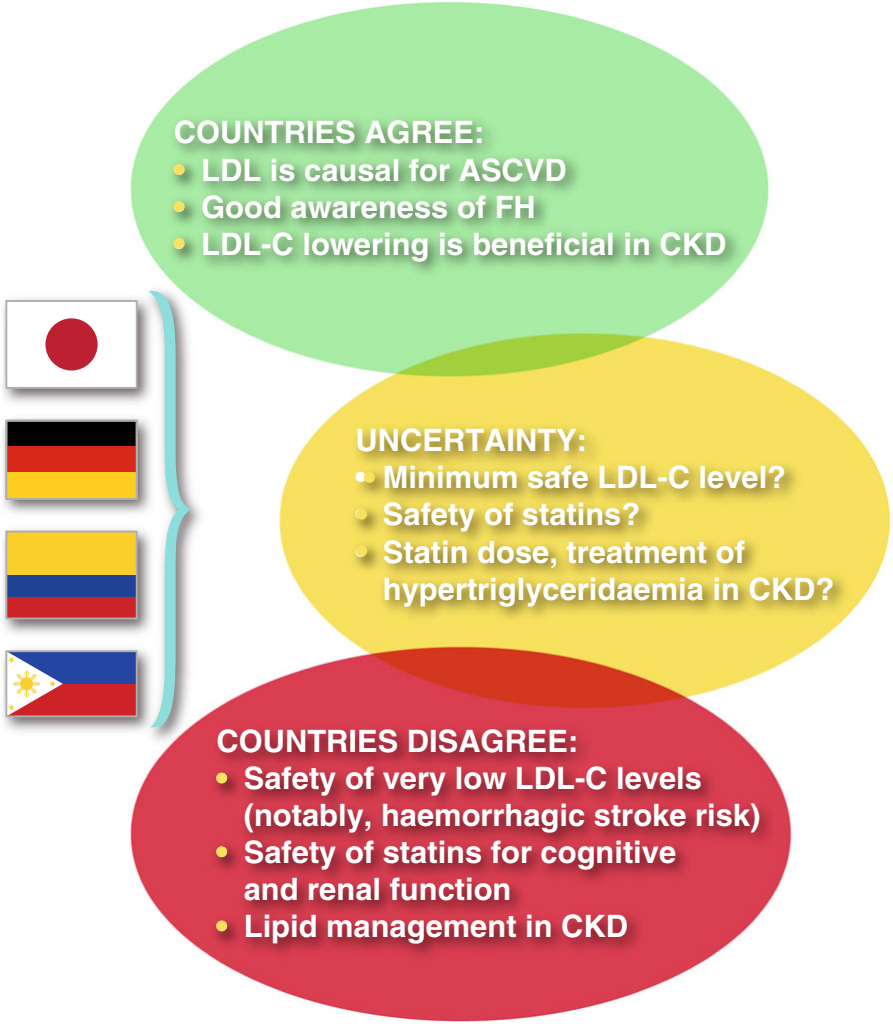
Summary figure: areas of agreement, uncertainty and disagreement in dyslipidaemia management between Japan, Germany, Colombia and the Philippines, based on a web-based survey of 1758 physicians

This survey has identified gaps in knowledge and behaviour amongst physicians managing dyslipidaemia in Japan, Germany, Colombia and the Philippines (Summary figure (Fig. 3)).

It is reassuring that there is almost universal agreement across all four countries regarding the causality of LDL-C in ASCVD. In addition, there is evidence of improvement in FH awareness and care, possibly reflecting a renewed focus from expert consensus groups [12, 13]. This builds on findings from the initial physician survey in Japan, which highlighted underdiagnosis of FH, especially among general practitioners, as an issue [11]. Despite the availability of well-developed evidence-based guidelines for dyslipidaemia management in each country or respective world region, areas of disagreement/uncertainty were identified [14–17]. These related to the safety of statin therapy, the safety of very low LDL-C levels, and dyslipidaemia management in CKD.

Consistent with the initial survey of physician attitudes in dyslipidaemia management in Japan [11], there were concerns regarding the safety of very low LDL-C levels attained on a statin (with or without other lipid modifying therapy). In the current report, these primarily focus on cognitive and renal function, as well as the risk of haemorrhagic stroke. While there is compelling evidence that a very low level of LDL-C may increase the risk of haemorrhagic stroke [18], especially in Asians [19], it is pertinent that there was no increase in risk at very low LDL-C levels attained by adding ezetimibe [3] or a PCSK9 inhibitor [4, 5] to background statin therapy in major clinical trials. Moreover, the well-documented reduction in the risk of ischaemic stroke in individuals who attain very low LDL-C levels outweighs any possible increase in the risk of a haemorrhagic stroke [2–4, 20]. With respect to cognitive adverse effects, the EBBINGHAUS study specifically investigated the effect of very low LDL-C levels using a computerised battery of tests for a range of cognitive domains including episodic and working memory, executive function, psychomotor speed and attention. This study showed no change in cognitive function even at very low LDL-C levels (0.28–0.44 mmol/L or 11–17 mg/dL) attained with the addition of a PCKS9 inhibitor [21]. It should be noted, however, that the duration of treatment with a PCSK9 inhibitor in this trial was relatively short and that longer-term data are still needed. There is no convincing evidence that the use of statins or attainment of a low LDL-C level causes renal dysfunction [2, 8]. Indeed, subgroup analyses from several major studies suggest that statins may have a renoprotective effect which merits further study [22].

Management of dyslipidaemia in CKD patients was another area of uncertainty in all four countries. There was a lack of consensus regarding the perceived need to reduce the statin dose in patients with CKD, the need for different LDL-C target levels depending on the stage of the CKD, as well as the clinical benefit of treating hypertriglyceridaemia in CKD patients.

The authors recognise several limitations relating to the methodology of this survey. First, physicians were recruited to the survey using a variety of approaches, including random selection from existing databases, contact via email and/or telephone, and recruitment from expert-led workshops. Second, the survey was self-report. Another potential source of relates to the distribution of specialties in the countries. Whereas in Japan and Germany there was an equal distribution across all specialties, in both Colombia and the Philippines, about half of all physicians were general physicians (46 and 55%, respectively). Thus, the study findings may have been influenced by differences in the knowledge base between different specialties. Indeed, this was evident in the initial survey conducted in Japan, in which cardiologists were shown to treat LDL-C more aggressively than those in other specialties or in general practice. Results from the PERCRO-DOC survey of more than 1300 randomly selected physicians in Croatia also indicated differences in the approach to cardiovascular prevention between general practitioners and hospital specialists. General practitioners were less likely to refer to guidelines compared with cardiologists and internists [23].

The extent to which the findings from this survey can be extrapolated to other countries is uncertain. Despite this, the results provide several important ‘take-home’ messages for physicians. In all four countries, there are key gaps in beliefs and practice that contribute to a roadblock for treatment of dyslipidaemia in people at high risk of ASCVD [10]. This was also evident in the initial physician survey; while most physicians (~ 80%) believed they treated dyslipidaemia appropriately, only about half (53.3%) knew the LDL-C target for high-risk patients. Moreover, only about half recognised the level at which high-density lipoprotein cholesterol was a marker of increased risk [11]. Similar findings were reported in the PERCRO-DOC survey [23]. Importantly, retrospective analysis of LDL-C goal attainment in more than 4000 outpatients in Italy in a real-world setting showed that less than 10% of patients considered at high or very high risk attained guideline-recommended LDL-C goals [24]. Real-world data derived from the Cegedim Longitudinal Practice Database in Germany, as well as observational findings from 18 countries outside Western Europe, also indicated inadequate use of lipid lowering therapy in high and very high-risk patients [25, 26].

Taken together, these findings highlight the need for educational programmes to increase awareness of the current evidence base supporting dyslipidaemia management. Where evidence is lacking, research is needed to address uncertainties, as well as education to put unresolved issues in perspective. Comparable surveys are also needed in North America, Eastern Europe and the Middle East to extend knowledge and improve dyslipidaemia management across all world regions.

## Conclusions

In conclusion, this survey provides a ‘snapshot’ of the beliefs and behaviour of physicians in Japan, Germany, Colombia and the Philippines who are involved in managing patients with dyslipidaemia in their routine practice. The findings highlight key areas of need for further education and research. The results provide a rationale for similar surveys in other countries, as well as follow-up surveys to assess the impact of any educational programmes and activities on these gaps in belief and practice in dyslipidaemia management.

## Data Availability

The datasets used and/or analysed during the current study are available from the corresponding author on reasonable request.

## Abbreviations

ASCVD: Atherosclerotic cardiovascular disease
CKD: Chronic kidney disease
FH: Familial hypercholesterolaemia
LDL-C: Low-density lipoprotein cholesterol
PCSK9: Proprotein convertase subtilisin/kexin type 9
